# Estimating preharvest density, adult sex ratio, and fecundity of white‐tailed deer using noninvasive sampling techniques

**DOI:** 10.1002/ece3.8149

**Published:** 2021-09-28

**Authors:** Jon E. Brommer, Jenni Poutanen, Jyrki Pusenius, Mikael Wikström

**Affiliations:** ^1^ Department of Biology University of Turku Turku Finland; ^2^ Natural Resources Institute Finland Joensuu Finland; ^3^ Finnish Wildlife Agency Helsinki Finland

**Keywords:** fecal DNA, Spatial Capture, Spatial Capture Recapture, white‐tailed deer, wildlife camera, wildlife management

## Abstract

Adult sex ratio and fecundity (juveniles per female) are key population parameters in sustainable wildlife management, but inferring these requires abundance estimates of at least three age/sex classes of the population (male and female adults and juveniles). Prior to harvest, we used an array of 36 wildlife camera traps during 2 and 3 weeks in the early autumn of 2016 and 2017, respectively. We recorded white‐tailed deer adult males, adult females, and fawns from the pictures. Simultaneously, we collected fecal DNA (fDNA) from 92 20 m × 20 m plots placed in 23 clusters of four plots between the camera traps. We identified individuals from fDNA samples with microsatellite markers and estimated the total sex ratio and population density using spatial capture–recapture (SCR). The fDNA‐SCR analysis concluded equal sex ratio in the first year and female bias in the second year, and no difference in space use between sexes (fawns and adults combined). Camera information was analyzed in a spatial capture (SC) framework assuming an informative prior for animals’ space use, either (a) as estimated by fDNA‐SCR (same for all age/sex classes), (b) as assumed from the literature (space use of adult males larger than adult females and fawns), or (c) by inferring adult male space use from individually identified males from the camera pictures. These various SC approaches produced plausible inferences on fecundity, but also inferred total density to be lower than the estimate provided by fDNA‐SCR in one of the study years. SC approaches where adult male and female were allowed to differ in their space use suggested the population had a female‐biased adult sex ratio. In conclusion, SC approaches allowed estimating the preharvest population parameters of interest and provided conservative density estimates.

## Graphical abstract

Wildlife, such as these white‐tailed deer, can be studied using noninvasive methods such as pictures taken with wildlife cameras or DNA extracted from feces picked up from the forest floor. Such noninvasive methods allow studying wildlife populations with minimal disturbance but analyzing such data has its limitations and challenges. In this study, we compare the kind of insights and information we can obtain from these two approaches.

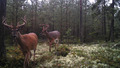

## 1 INTRODUCTION

Sustainable management of game animals requires knowledge of their population densities, as well as of key markers of population performance such as adult sex ratio and fecundity (Caughley & Sinclair, 1994). When a population is harvested, its sex and age ratio is impacted with the extent depending on the hunting regulations and local practices. In Nordic countries, for example, large ungulates are regulated via hunting license practices aimed to harvest primarily young animals and adult males, resulting in high proportion of females with high reproductive output (Langvatn & Loison, 1999; Sæther et al., 2004). Obtaining estimates of adult sex ratio and fecundity from free‐ranging populations is not trivial as it requires estimating densities of at least three classes of animals in the population: adult males, adult females, and juveniles.

Wildlife cameras provide a cost‐efficient approach to obtain encounter (i.e., detection/nondetection) information on wildlife (Burton et al., 2015; Sollman, 2018). In many species, adult males, females, and juveniles can be distinguished in pictures, thereby opening up the possibility to infer several key demographic properties of a population. Furthermore, the lower cost of wildlife cameras makes them amenable for studies on larger spatial scales, for example, in citizen science projects (Steenweg et al., 2017). Whereas earlier studies routinely were based on the raw count data of pictures or videos that wildlife cameras collect, it has become clear in the last decade or so that proper use of information from wildlife cameras requires statistical analyses for which various approaches are possible (Burton et al., 2015; Dénes et al., 2015; Sollman, 2018). When individuals are identifiable from camera pictures, a group of cameras may provide spatially explicit capture–recapture information on individuals in a noninvasive manner. Such data can be analyzed using spatial capture–recapture (SCR) models to provide information on density and space use (Efford, 2004; Efford & Fewster, 2013; Royle, Richard, et al., 2013). The SCR approach assumes animals have a activity center where their probability to be detected by the camera or other “trap” is maximal. This detection probability then declines with increasing distance between the activity center and the trap assuming a specific function that depends on the space use. Density is then the number of activity centers in what is termed the state space (area covered by the traps and a certain buffer). As the SCR approach is spatially explicit, its basic implementation can be extended with geographical information to provide insights in general ecology of the species including resource selection (Royle, Chandler, et al., 2013) and landscape connectivity (Sutherland et al., 2015). The SCR approach can furthermore readily integrate information obtained using complementary approaches such as GPS or radio‐tracking location data (Royle, Richard, et al., 2013).

When individuals cannot be identified from the pictures, spatial capture (SC) is one possible alternative (Chandler & Royle, 2013) for analyzing the data. The SC method is also referred to as “unmarked SCR” (Johnson, 2019) or “spatial correlated count” (Burgar et al., 2018). SC is essentially an SCR approach and hence assumes the same parameters as SCR, except it only requires information on total counts of the animals at each camera trap instead of individual‐specific counts (Chandler & Royle, 2013). Among other alternative approaches also accounting for imperfect detection (Dénes et al., 2015), SC stands out by inferring the density on the basis of the spatial correlation expected in counts made at locations sufficiently close to each other for individuals to move between them (Chandler & Royle, 2013; Ramsey et al., 2015). For example, when a group of cameras are placed such that the distance between them is within the home‐range area of the focal species, the spatial correlations arise because the same individuals are potentially recorded at multiple cameras. Because inferring density on the basis of count data alone is highly demanding, the SC approach requires prior or auxiliary information, typically on the space use of the target species (Chandler & Royle, 2013; Ramsey et al., 2015).

Other approaches to infer density from wildlife camera pictures that do not require individual identification include approaches based on animal movement characteristics (Random Encounter Model, Rowcliffe et al., 2008) and analyses based on distance sampling (Howe et al., 2017; Rowcliffe et al., 2011) and time‐to‐encounter models (Moeller et al., 2018) that of course come with assumptions of their own (for an overview, see Sollman, 2018). Whereas SC analyses require only a count of animals in each picture, the random encounter model and distance sampling require additional interpretation of pictures. In particular, they require inferring the distance of the animal from the camera and, depending on the camera setting, analyzing series of pictures recording the same animal (e.g., to infer movement speed and to avoid pseudoreplication). In addition, these approaches are design‐based (as opposed to model‐based SCR/SC) and are less flexible incorporating model violations.

**FIGURE 1:**
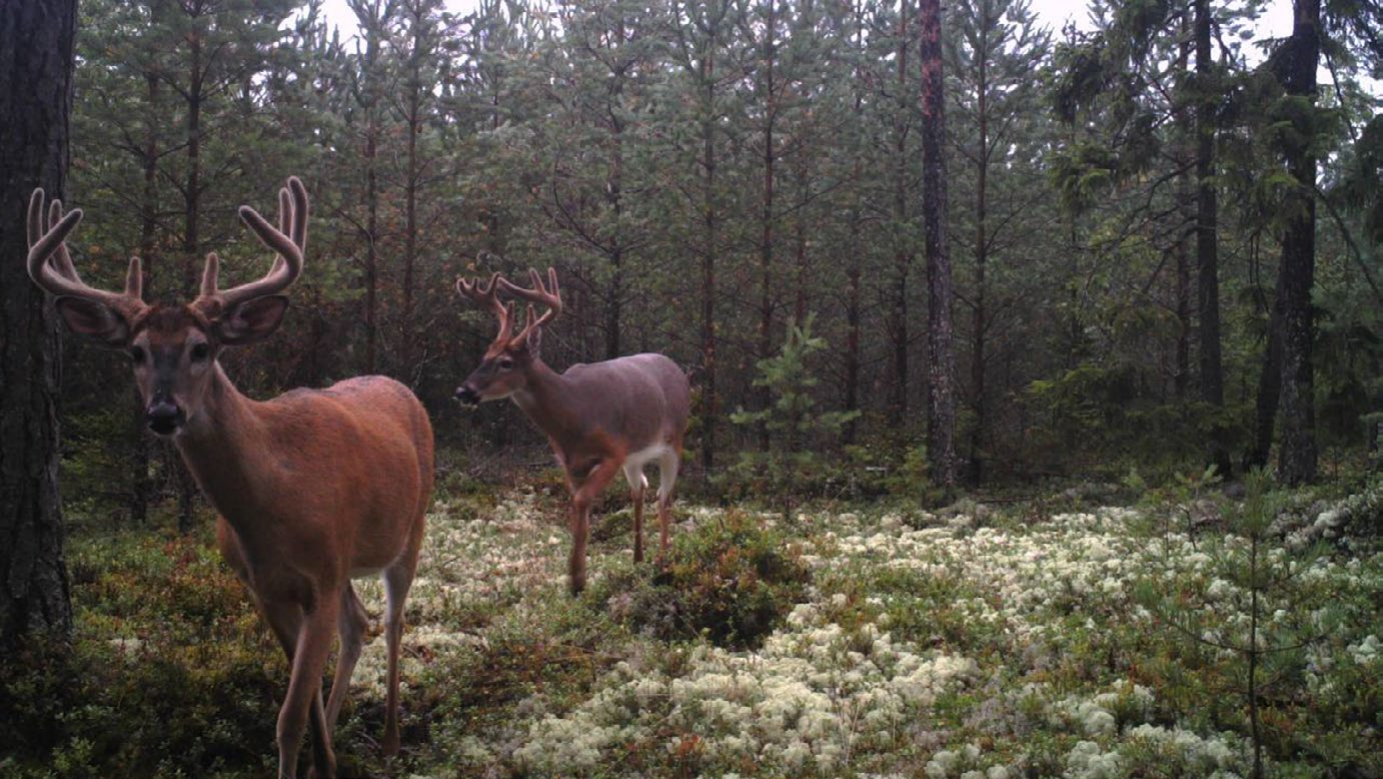
Two male white‐tailed deer caught on camera during this study

Here, we study the potential of wildlife cameras to infer preharvest density of white‐tailed deer (Figure 1) in southern Finland using an SC approach. Our objective is to infer both sex and age (juvenile vs. adult) classes within a “snap‐shot” setting of two to three weeks under which the population closure assumption (no births, deaths, emigration, or immigration) is likely to hold. By using cameras in late summer, fawns (juveniles) and female and male adult white‐tailed deer can be readily distinguished. During this time of the year, adult males carry antlers, and fawns are large enough to move around to be detected by wildlife cameras, and can be identified as juveniles as they typically still have a spotted pelage and are also smaller in size than adult females. As a consequence, the required interpretation of pictures is restricted to counting the animals in these three age and sex classes (adult males, adult females, and fawns) for each picture. This level of picture interpretation requires minimal training of personnel or picture analysis software for automated interpretation and is anticipated to scale up readily. Furthermore, by studying the population prior to harvest, its density is at its annual peak which likely facilitates obtaining sufficient detections for analysis.

Inferring density of white‐tailed deer fawns and females in an SC setting requires additional information, for example, on the movement of individuals during the study period (Ramsey et al., 2015). In ungulates (and many other mammals), adult males typically have larger home ranges than females (white‐tailed deer: Lesage et al., 2000, Dechen Quinn et al., 2013, Honzová, 2013). Our study was conducted prior to the rut (which is in November); adult males increase their daily movements markedly during the rut (Webb et al., 2010). Juvenile white‐tailed deer in their first 2 months are still heavily dependent on their mothers and are relatively inactive with very small home ranges during this time, but become after this period rapidly more semi‐independent (Hiller et al., 2009). Even though home ranges are well described for many populations, and thus, literature estimates are available, space use can still differ strongly between sites. For this reason, application of SC benefits greatly from collecting telemetry information on a subset of individuals during the study using, for example, GPS collars (Furnas et al., 2018). Nevertheless, auxiliary information on space use can be expensive to collect and typically require invasive methods. In this study, we explore three noninvasive approaches for providing information of the space use of the white‐tailed deer.

First, to provide information on space use as well as an estimate of total density of white‐tailed deer, we collect noninvasive fecal DNA samples for individual identification simultaneously with the wildlife camera survey. DNA‐based individual identification allows SCR to be conducted (Royle, Richard, et al., 2013). When inferring density prior to harvest in early autumn, both adults and juveniles are present in the population, but because DNA does not allow aging of individuals, the approach is unable to distinguish these age groups. Hence, DNA‐based SCR presents a kind of weighted average across fawns and adults for each sex in the study population. Furthermore, SCR analysis of fecal DNA provides an estimate of population density, which is independent from estimates derived from wildlife camera data. Second, we use literature‐based values of space use of white‐tailed deer in Finland. Honzová (2013) reported monthly home‐range areas of male and female white‐tailed deer that were fitted with a GPS collar. Collared white‐tailed deer were of different age classes and tracked in several sites across Finland, and hence are not specific to our study population. It is, however, the only published statistics that we are aware of, that is of most relevance to our study population. Third, the white‐tailed deer males can be individually identified on the basis of their antler characteristics, which allows SCR analysis. Thus, we can from the wildlife camera data itself infer space use and density of adult males in the population using SCR and combine this in one model with SC analysis on adult females and fawns.

Our study question is whether these SC approaches can provide reasonable inferences of density, adult sex ratio, and fecundity given what we know about these demographic parameters in white‐tailed deer. We collected camera trap data during a short‐term (2–3 weeks) period repeated in two years (2016, 2017), where we simultaneously collected fecal sampled. We used fecal DNA‐based individual encounter histories analyzed in a spatial capture–recapture (SCR) framework for comparison with inferences made using SC on camera trap data.

## 2 MATERIALS AND METHODS

### 2.1 Study area

The study area (60°52′7″N, 22°49′13″E (WGS84)) was situated in a landscape typical for southern Finland. The landscape is a mixture of fields and forest in approximately equal proportion. Forest patches consist mainly of coniferous dominated tree species (spruce *Picea abies* and pine *Pinus sylvestris*) or then mixed with deciduous trees (birch *Betula* spp. and aspen *Populus tremula*). The study area was not intersected by any road or larger trail.

### 2.2 Fecal DNA sample collection

**FIGURE 2:**
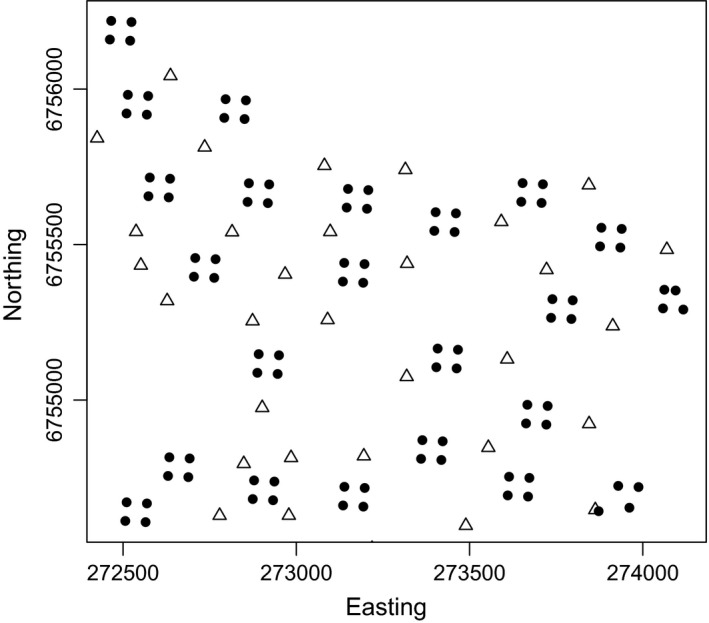
Simple plot of the spatial layout of the cameras (triangles, *n* = 36) and fDNA sample plots (dots, *n* = 92) in 2016. Locations were approximately the same in 2017, but one camera failed to operate. Northing and easting are provided in ETRS89‐TM35FIN (EPSG:5048) coordinates whose unit is meters

Fecal DNA (fDNA) was sampled in 92 sample plots in both study years. Each plot was 20 m × 20 m in size and marked in the field with ribbons. Plots were grouped in cluster following the design advocated by Sun et al. (2014). Earlier work on fDNA‐based SCR in white‐tailed deer in Finland (Poutanen et al., 2019) based on clustered sample plots included simulations that suggested that for a study period of 2–3 weeks, the spacing between clusters should be about 250 m. We here spaced clusters at about 300 m distances. We used 23 clusters of four plots. The four plots in a cluster were placed in a square with their center coordinates 60 m apart. Legal restrictions on placing cameras in planted fields prohibited a strict regularity of the grid (Figure 2). Plots were emptied of all fecal pellets on the first visit. Setting up all fecal sample plots took two days. In 2016, sample plots were visited with weekly (7 days) interval for 2 occasions after the initial cleaning visit, hence covering a total period of 16 days (setting up and sampling the first half the plots on days 1, 8, and 15; second half on days 2, 9, and 16). In 2017, sample plots were visited with four‐day interval for 5 occasions after the initial cleaning, hence covering a period of 22 days (setting up and sampling the first half of the plots on days 1, 5, 9, 13, 17, and 21; second half on days 2, 6, 10, 14, 18, and 22). The two consecutive days of sampling that it took to cover all plots was considered as one occasion and the time interval between occasions was hence 7 and 4 days for 2016 and 2017, respectively. In general, more occasions and shorter time intervals between occasions improve SCR model inferences (Royle, Richard, et al., 2013), but was not possible in both years. At each visit to a plot except the first (cleaning) visit, few fecal pellets were sampled from each pellet group in a resealable plastic bag after which all remaining pellets were removed from the plot. Samples were frozen at −20°C until further analysis.

### 2.3 DNA extraction and identification of individuals

We followed the protocol of Poutanen et al. (2019) for DNA extraction and individual identification. A minor modification for the microsatellite PCR protocol was that the final concentration of primer Rt5 was decreased to half (0.1 μmol/L) and BSA concentration to one‐tenth (0.1 μg/μl) of the original concentration. Briefly, 14 microsatellites were used and at least three PCR replicates were performed for each DNA sample. Based on Cervus 3.0.7 (Kalinowski et al., 2007), the observed probability of identity between siblings (PIDsib) using seven least informative loci was low (<0.005) and follows the recommendations (Waits et al., 2001). We allowed a maximum of two mismatches in different loci between the genotypes in order to being matched to same individual. Therefore, if 11 or more loci were amplified, we used the sample in identification analysis. The rule for constructing the final consensus genotype based on at least three replicate runs was that for each locus the consensus is a homozygous locus if the alleles of homozygous loci were amplified three times and the consensus is a heterozygous locus if the heterozygous loci amplified two times. At least one DNA sample of each identified individual was sexed with X and Y chromosome‐specific primer pair ZFX/ZFY. Based on results of Poutanen et al. (2019), we assumed that the 11 to 14 microsatellite markers used here were sufficient to exclude possible roe deer DNA from further analyses.

### 2.4 Camera data collection

A total of 36 trail cameras (Uovision UV595) were placed in the study area. This camera uses passive infra‐red (PIR) detection of motion and has an infra‐red flash for night pictures. Trail cameras were placed at approximately 300 m distances between adjacent cameras in between fecal DNA sample clusters (Figure 1). Cameras were set to take bursts of 3 pictures when triggered with a five‐second delay to being potentially triggered again. Cameras were operational before the period in which fecal DNA was sampled but we here use pictures recorded during the same sampling period as fDNA was sampled (i.e., 14 days in 2016; 20 days in 2017).

All obtained pictures were interpreted by a human (JP (author Jenni Poutanen 2016, research assistant 2017) scoring for each picture the number of fawns, adult male, and adult female white‐tailed deer. The pictures of the white‐tailed deer where the sex or age could not be identified were categorized as white‐tailed deer of unknown class and were discarded from further analyses (approximately 7.5% of pictures containing white‐tailed deer). After this screening, males were identified from all the pictures of males on the basis of their antlers (JP in 2016, JP and research assistant in 2017). When there were two observers (2017), male identity was only assigned if both were in agreement. Lastly, author JP again evaluated the male identity assignments of 2017 data paying special attention to putative male individuals that were recorded at different cameras. Pictures where the male individual could not be reliable identified were classified as “unknown adult male.”

### 2.5 Encounter data

Wildlife cameras with the above‐described settings produce many consecutive pictures that are nonindependent as it is likely that the same individual triggers the camera multiple times. We assumed that pictures taken by a camera within 1 hr are all potentially from the same individual(s). All pictures where the time interval between consecutive pictures was less than 1 hr were therefore grouped into what we here term “encounter event.” For each encounter event, the numbers of adult females, adult males, and fawns were inferred to be the maximum number of females, males, and fawns that could be counted in one picture taken during each encounter event (not necessarily the same picture for each class). As the wildlife cameras are active over several days, we needed to group pictures in presumably independent occasions of recording. We considered each 24‐hr period (from midnight to midnight) as one occasion.

### 2.6 fDNA‐SCR model in secr

We fitted a standard likelihood‐based SCR model (e.g., Efford & Fewster, 2013) on the individuals identified from fDNA using secr (Efford, 2018) package implemented in R (R Core Team, 2018). We used the central location of each fDNA sample plot as its spatial coordinate. In secr terminology, fDNA sample plots are “proximity detectors.” The SCR model implemented estimates the detection at the activity center (*g*_0_) and assumed that the decline in detection probability with distance followed a half‐normal function specified by the space‐use parameter *σ* (Efford, 2018). The state space consisted of the locations of the fDNA sample plots buffered by 2000 m. The chosen buffer was larger than the buffer suggested by the diagnostics of secr (such as suggest.buffer and esa.plot), but was used to keep the same state space in all analyses including the SC analyses. We compared various candidate models based on their AICc values. Sex of the individual was included as a hybrid mixture in all models (Efford, 2018). Candidate models considered included various combinations of covariates for the detection probability parameter g_0_ and space‐use parameter *σ*, where both parameters could be (1) constant, (2) sex‐dependent, as well as that g_0_ could be (3) occasion‐specific, (4) show a behavioral response (i.e., detection in a trap changes after an individual has been encountered once in that trap).

We used secrdesign (Efford, 2019) to evaluate bias and precision of estimates of density and *σ* given our spatial layout of sampling plots. We used as simulation parameters secr‐derived estimates of *g*_0_, density and *σ* obtained under the top model. Based on these parameters, secrdesign simulated 250 data sets to be analyzed using secr. Relative bias [the error (difference between inferred and simulated value) divided by the simulated value] and precision [computed as root mean square error (RMSE)] were computed. We thus performed simulation analysis to evaluate the performance of our specific design (i.e., location of sample plots, number of sampling occasions). Through simulations, we investigated how increasing the number of sampling occasions affected relative bias and RMSE. In case our sampling was insufficient, increasing the number of sampling occasions causes a reduction in relative bias.

### 2.7 Spatial capture analysis of camera data

Camera traps are placed at fixed points in space. The state space considered in the model is the boundary box of the camera traps surrounded by a buffer area. Each camera can record same individuals multiple times. Pictures of white‐tailed deer were classified into 3 groups (*g*): adult males (*m*), adult females (*f*), and fawns (*c*). We used data augmentation (Royle, Richard, et al., 2013), assuming there were a maximum of *M*_m_, *M*_f_, and *M*_c_ for the groups males, females, and fawns, respectively, in the state space. We assumed the latent state for individual *i* belonging to group *g* to be present in the state space was

(1) $$z_{\mathrm{ig}}∼\text{Bernoulli}\;\left(\psi_{g}\right),$$

where $\psi_{g}$ denotes the probability for an individual belonging to group *g* to be in the state space ($z_{\mathrm{ig}}=1$) or not ($z_{\mathrm{ig}}=0$). The abundance of animals belonging to group *g*, *N_g_*, hence is obtained by summing over all *z_ig_*. Data augmentation hence requires that *M* > *N* and in practice it is important that *M* is greater than the maximal posterior value of *N* (Royle, Richard, et al., 2013). If it is in the state space, individual *i* belonging to group *g* can be observed at camera trap *j*. Assuming that the number of observations *y* of individual *i* belonging to group *g* at camera trap *j* is Poisson distributed, we can model this number of observations over all *K_j_* occasions that trap *j* was active as

(2) $$y_{\mathrm{igj}}∼\text{Poisson}\;\left(K_{j}\lambda_{\mathrm{igj}}\right),$$

where $\lambda_{\mathrm{igj}}$ is the encounter rate of individual *i* belonging to group *g* at camera trap *j*. We assumed the encounter rate was Gaussian bivariate distributed around individual *i*'s activity center **s** *_i_*, such that for trap *j* located at **x** *_j_* in space

(3) $$\lambda_{\mathrm{igj}}=\lambda_{0g}\exp\left(\frac{‐{∥x_{j}‐s_{i}∥}^{2}}{2\sigma_{g}^{2}}\right)z_{\mathrm{ig}},$$

where $∥x_{j}‐s_{i}∥$ denotes the Euclidean distance between activity center and trap location, $\lambda_{0g}$ is the baseline detection probability for group *g*, and $\sigma_{g}$ a group‐specific parameter which scaled how rapidly detection drops as the trap is placed further from the activity center of each group. The activity centers **s** are latent variables, which are, by definitions, placed in the state space considered. These equations are central to spatial capture–recapture (SCR) models (Royle, Richard, et al., 2013) and are hence applicable when individual *i* can be identified.

When individual identification is not possible, the total number of adult males (*m*), adult females (*f*), and fawns (*c*) can still be counted, because these groups can be readily distinguished on the pictures. Thus, without individual identification the available encounter history is trap‐specific total of animals belonging to the various groups *g* encountered. These totals are, conceptually, the result of summing up over all latent observations *y_igj_* (Equation 1) (Chandler & Royle, 2013). That is, the total number of observations in trap *j* for each group *g* over all *K* occasions that trap *j* was active is

(4) $$n_{\mathrm{jg}}∼\text{Poisson}\;\left(K_{j}\Lambda_{\mathrm{jg}}\right),$$

where $\Lambda_{\mathrm{jg}}$ represents the summed up encounter rate for every camera trap *j* over all individuals *i* belonging to group *g* which are observable in the state space, such that

(5) $$\Lambda_{\mathrm{jg}}=\lambda_{0g}\sum_{i=1}^{M_{g}}\exp\left(\frac{‐{∥x_{j}‐s_{i}∥}^{2}}{2\sigma_{g}^{2}}\right)z_{\mathrm{ig}}.$$

In terms of data obtained, for each camera trap *j*, $n_{jc.}$ and $n_{jf.}$ and $n_{jm.}$ are the sums of fawns, adult females, and adult males, respectively, over all encounter events.

The above formulation assumes homogeneity in encounter probability and encounter rate across occasions and cameras, as well as homogenous density across the state space. All of these assumptions can be relaxed by adjustment of the above outlined basic model formulation as detailed in Royle, Richard, et al. (2013). However, as parameters in the SC model are inferred only on the basis of counts, which is demanding, more complicated model formulations were not attempted.

Inferences on the number of individuals present in the state space can be derived from the latent states. In particular, the total population size of individuals of group *g* in the state space was

(6) $$N_{g}=\sum_{i=1}^{M_{g}}z_{\mathrm{ig}}.$$

Adult sex ratio was calculated as the ratio of adult males to the sum of adult males and females in the state space, $N_{m}/\left(N_{m}+N_{f}\right)$ and fecundity as the ratio of fawns to females in the state space $N_{c}/N_{f}$. Density is the number of individuals in the state space divided by the size of the state space and was expressed as individuals per km^2^.

### 2.8 SCR on camera data for males combined with SC on camera data for females and fawns

The above model formulation can consider two subsets of a single population; individually identifiable animals (marked individuals) and those that cannot be individually identified (unmarked individuals). In our case, we consider a model formulation in which males are considered as marked (individually identified from pictures by their antlers). For the model formulation in which males are considered marked, we calculated for each male individual *i* the total number of encounter events it was recorded in trap *j* over all *K* occasions, $y_{\mathrm{imj}}$ (Equation 2). However, it was not always possible to identify each male on the basis of the characteristics of the antlers due to movement or incomplete view of the antlers, resulting in recordings of unidentified males. Ignoring such unidentified males will downward bias the encounter rate for males. We therefore incorporated a correction factor *p*_ID_ following (Royle, Richard, et al., 2013, p.514) and modified Equation (2) by assuming that the number of encounter events for individual *i* belonging to group *m* (male) in trap *j* was

(7) $$y_{\mathrm{imj}}∼\text{Poisson}(K_{j}\lambda_{\mathrm{imj}}p_{\text{ID}}),$$

where $\lambda_{\mathrm{imj}}$ denotes the encounter rate for individual *i* belonging to group *m* (male) in trap *j* (as given by Equation 3), *p*_ID_ is the probability that a male is identified individually, *K_j_* the number of occasions trap *j* was active. The probability *p*_ID_ was assumed to be related to the total number of recorded males *n*_TOT_ and the number of identified males *n*_ID_ as

(8) $$n_{\text{ID}}∼\text{Binomial}\left(p_{\text{ID}},n_{\text{TOT}}\right).$$

That is, we assumed no spatial or temporal heterogeneity in this probability. More complete approaches that also use the spatial information of identified and nonidentified individuals have been employed (Jiménez et al., 2019). The primary purpose of our procedure is to provide unbiased estimates of the encounter rate for males such that it reflects all males (both identifiable and nonidentifiable).

### 2.9 Implementation of the SC and SC/SCR models

All SC models were implemented in JAGS (Plummer, 2003). Priors on **s** are uniformly distributed throughout the state space. We used beta(1,1) priors on all ψ, and uniformly distributed priors on *λ* (0,5). Priors for *p*_ID_ was beta(1,1). The SC model requires informative priors (Chandler & Royle, 2013), and we consider here three versions with the following acronym and description:

1. SC‐fDNA. The prior for the movement parameter sigma (σ) of adult males, adult females, and fawns is the sigma inferred in our analysis of fDNA‐based SCR specific to the year studied. Thus, the prior for sigma in the SC model for 2016 is the sigma estimated by SCR on the basis of the 2016 fDNA data, and likewise for 2017.
2. SC‐lit. The prior for the sigma of adult males, adult females, and fawns is the sigma inferred from literature. Movement data obtained from GPS‐collared white‐tailed deer in Finland showed that the 95% usage of the monthly home‐range area was approximately 156ha and 733ha per month for females and males, respectively, in autumn (Honzová, 2013). Using an approximation based on the chi‐square distribution and assuming the area used is bivariately normal (Royle, Richard, et al., 2013, p. 136),

(9) $$\sigma =\sqrt{\frac{A_{0.95}}{\chi_{2}^{2}\left(\mathrm{Pr}=0.95\right)\pi }}=\sqrt{\frac{A_{0.95}}{5.99\pi }},$$

where *A*_0.95_ is the area with 95% probability to be used around the activity center, $\chi_{2}^{2}\left(\mathrm{Pr}=0.95\right)$ is the chi‐square value for 2 degrees of freedom at 95% probability. From this approximation, it follows that a naïve estimate of the parameter *σ* in the bivariate normal detection function was 287 m and 624 m for females (assumed identical for fawns) and males, respectively. This movement parameter is for the time period of one month (30 days) but we here implement these values for each of the study years despite the fact that we collected data during time periods shorter than one month. Hence, this literature‐based estimate of the parameter *σ* may present an overestimate of the movement during the study period.

- SC‐SCR. The prior for adult male sigma was uninformative (uniform in the range of 0 m to 2,000 m) as the posteriors for male sigma were inferred using males identified from camera pictures in SCR analysis as explained above. For adult females and fawns, we assumed an informative prior set at fDNA‐SCR inferred sigma which was considered the most relevant parameter as it was specific to the study area and year.

Given the Gaussian distribution of sigma assumed in the source method (home range or fDNA‐SCR), we assumed a normal distribution for the informative prior of sigma around its point value (as detailed above) with a variance chosen such that the distribution adhered to the source. That is, for sigma based on fDNA, the variance matched the confidence interval assuming the entire confidence interval was approximately four times the square root of the variance (i.e., four times the standard error under assumption of a Gaussian distribution). For the literature estimate, the variance matched the uncertainty in the estimate presented (males: 5,000; females: 1,000). Adapting and burn‐in were 1,000 and 4,000 iterations, respectively. The length of the posterior samples was adjusted to make sure the Monte–Carlo error was below 5% of the standard deviation of each parameter, and that posterior chains exhibited low autocorrelation and good mixing (R‐hat below 1.1 for all parameters). These criteria resulted in either 1,000 or 1,500 posterior samples drawn of each of 3 chains after thinning 20.

### 2.10 Data and script accessibility

Data and JAGS script for the SC analyses of all three models as well as the data for the fecal DNA SCR are available from the Dryad data repository associated with this publication.

## 3 RESULTS

### 3.1 SCR analysis of fDNA data

In total, 32% of the samples were successfully genotyped to the level permitting individual assignment. In 2016, we carried out 2 sampling occasions with a week interval and obtained individual encounters of 12 identified males (10 encountered once (not recaptured) and 2 three times) and 26 females (9 encountered once (not recaptured), 5 twice, 9 three times, 1 four times, and 2 five times). Model comparison showed that the most parsimonious model for 2016 had sex‐specific detection probability where the detection probability for females was about fivefold that of males, but there was no clear evidence for sex‐specific space use (*σ* parameter) and the sex ratio was equal (Table 1).

**TABLE 1:** Comparison of spatial capture–recapture (SCR) models ordered according to their AICc value, with parameter estimates of the most parsimonious model for white‐tailed deer identified from fDNA in 2016 and 2017

Model	npar	logLik	AICc	dAICc	AICcwt	Top model			
						par	est	lci	uci
*2016*									
*g*_0_(sex)*σ*(·)	5	−294.82	601.5	0.0	0.4				
*g*_0_(*t*+sex)*σ*(·)	6	−293.83	602.4	0.8	0.3	*D* (km^−2^)	11.8	6.8	20.4
*g*_0_(sex)*σ*(sex)	6	−294.69	604.1	2.6	0.1	*g*_0_ (female)	0.117	0.075	0.176
*g*_0_(·)*σ*(sex)	5	−296.58	605.0	3.5	0.1	*g*_0_ (male)	0.024	0.008	0.066
*g*_0_(*t+*sex)*σ*(sex)	7	−293.70	605.1	3.6	0.1	*σ* (m)	305	245	381
*g*_0_(·)*σ*(·)	4	−300.69	610.6	9.1	0.0	Sex ratio	0.523	0.285	0.752
*g*_0_(*t*)*σ*(·)	5	−299.69	611.3	9.7	0.0				
*g*_0_(*b*)*σ*(·)	5	−300.67	613.2	11.7	0.0				
*2017*									
*g*_0_(*t*)*σ*(·)	8	−532.80	1,084.2	0.0	0.5				
*g*_0_(*t*+sex)*σ*(·)	9	−531.92	1,085.0	0.9	0.3	*D* (km^−2^)	23.3	17.4	31.2
*g*_0_(*t*+sex)*σ*(sex)	10	−530.93	1,085.9	1.7	0.2	*g*_0_ (1)	0.028	0.016	0.048
*g*_0_(·)*σ*(sex)	5	−540.14	1,091.3	7.1	0.0	*g*_0_ (2)	0.038	0.023	0.062
*g*_0_(·)*σ*(·)	4	−542.03	1,092.7	8.5	0.0	*g*_0_ (3)	0.020	0.010	0.037
*g*_0_(sex)*σ*(·)	5	−541.12	1,093.2	9.1	0.0	*g*_0_ (4)	0.061	0.039	0.093
*g*_0_(sex)*σ*(sex)	6	−530.14	1,093.7	9.5	0.0	*g*_0_ (5)	0.052	0.033	0.082
*g*_0_(*b*)*σ*(·)	5	−541.55	1,094.1	9.9	0.0	*σ* (m)	217	184	256
						Sex ratio	0.379	0.271	0.501

Note

Analyses carried out in secr. Presented for each model are the number of parameters (npar), the log‐likelihood (logLik), small‐sample AICc, the difference between each model and the AICc of the top model (dAICc), and the support (AICcwt). For the top model, the estimate (est), lower and upper confidence interval (lci and uci, respectively) are given for density (*D*) in individuals km^−2^, baseline detection probability *g*_0_, *σ* (in m) and the probability an individual is a male (sex ratio). Model variants concern different covariates placed on these parameters, either constant (·), sex‐specific (sex), occasion‐specific (*t*), or behavioral response (*b*). The top model estimates for *g*_0_ in 2016 are separate for female and male, and in 2017 separate for the five sampling occasions.

In 2017, in the same fDNA plots sampled over 5 occasions with four‐day interval, 41 females were identified (22 encountered once, 6 twice, 8 three times, 4 four times, 1 seven times) and 25 males (15 encountered once, 5 twice, 5 three times). The most parsimonious model for 2017 fDNA data included occasion‐specific detection probability, with support for occasion‐ specific detection, but again no clear support for sex‐specific *σ* (Table 1). Sex ratio in 2017 showed evidence of female bias. As expected for fDNA‐based encounters, a behavioral response was not supported (Table 1). Overall, the top models’ parameters for both years agreed reasonably well given that their confidence limits were overlapping.

**FIGURE 3:**
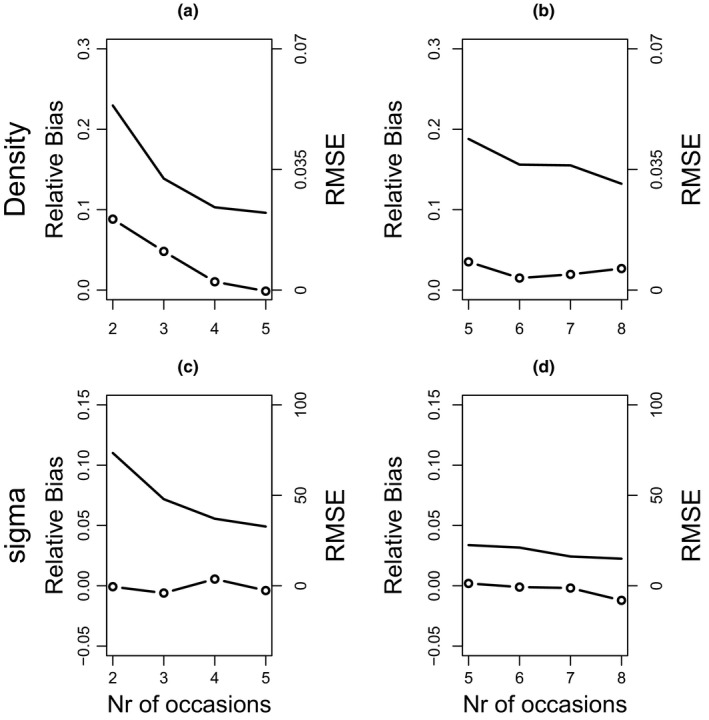
Relative bias (dots and lines) and root mean square error (RMSE, line) plotted for 250 a posteriori simulations of the fDNA‐SCR design for 2016 (a,c) and 2017 (b,d). Relative bias and RMSE are plotted for both density (a,b) and sigma (c,d). Simulation assumes that year‐specific density and sigma were as estimated by the top model (Table 2). Detection was conservatively assumed to be 0.03 in all simulations. In the design used, there were 2 occasions in 2016 with 7 days of interval, and 5 occasions with 4 days of interval in 2017

We conducted simulations to evaluate the bias and precision of our fDNA‐based sampling design. Simulations were based on the year‐specific density and *σ* of the top model (Table 2), and we assumed a constant detection probability *g*_0_ conservatively set at 0.03 for both study years (Table 2). Simulations suggested for 2016 that our design provided an unbiased estimate of *σ*, but that density was overestimated (Figure 3a,c). In particular, increasing the number of occasions from 2 to 5 (i.e., shortening the time between fecal sample collections from 7 days to approximately 3 days) would reduce relative bias in density from +8.8% to 0% (Figure 3a). In 2017, however, the relative bias in density for the used number of sampling occasions (5 occasions with 4 days interval) was only +3.4% and was largely unaffected by increasing the number of sampling occasions (Figure 3b) and that *σ* was estimated without bias.

**TABLE 2:** Description of the pictures collected in 2016 and 2017

	2016	2017
Number of cameras	36	35
Number of 24‐hr occasions	16	22
Pictures with white‐tailed deer	5,224	6,636
Encounter events (1‐hr grouping)	436	1,162
Encounter event/camera/day	0.76	1.5
Male individuals identified	7	17
All males/occasion/camera	0.066	0.184
Females/occasion/camera	0.34	0.47
Fawns/occasion/camera	0.53	0.47
Fawns/female	1.55	1.00

Note

In both years, cameras were operational in approximately three weeks (36 cameras for 16 days in 2016, 35 cameras for 22 days in 2017) and in total >10,000 pictures were taken. Adult males were individually identified on the basis of their antlers. An occasion consisted of 24 hr from midnight to midnight. Pictures taken within one hour of each other were grouped to what we here refer to as “encounter event” as these pictures likely represent repeated observations of the same animals.

### 3.2 Wildlife camera data

Camera traps collected a high number of pictures in both years; many of these pictures were putative duplicated records, and the number of encounter events (presumably independent recordings) was at most 1.5 encounter event per day per camera (Table 2). Relatively few encounter events contained an adult male compared with adult female or fawn (Table 2). Adult males could be individually identified each year on the basis of antler characteristics, although their numbers were restricted, especially in 2016 (Table 2). Fawns were detected at least as often as females, and the ratio of the number of pictures of fawns over the number of pictures of females (which can be considered as a naïve estimate of reproduction) exceeded 1 in 2016 but not in 2017 (Table 2).

### 3.3 SC analysis

Because the SCR analysis based on fDNA did not find support for *σ* differing between sexes (Table 1), we implemented SC analysis with informative prior for *σ*, being identical for each class (males, females, and fawns), at a year‐specific value (SC‐fDNA; Table 3). In addition, we implemented SC with priors for *σ*, where the estimate was specific for males and females (fawns’ identical to females’), informed by published estimates (SC‐lit; Table 4). Lastly, we implemented SC with priors for *σ* for fawns and adult females (identical) informed at the year‐specific values estimated by fDNA‐SCR, combined with SCR for adult males on the basis of pictures (SC‐SCR; Table 5). The latter SCR analysis indicated that adult males could be identified in about two‐thirds of the encounter events (*p*_ID_ in Table 5) and that adult male encounter rate *λ* was low, especially in 2017 (Table 5). The adult male *σ* as estimated by camera‐based SCR agreed well with the literature value (Table 5).

**TABLE 3:** Parameter estimates of the spatial capture analysis (SC) where an informative prior for *σ* was based on the year‐specific values inferred by fDNA‐SCR (see Table 1)

	2016			2017		
	Estimate	Lower	Upper	Estimate	Lower	Upper
*σ* (m)	303	300	313	167	147	187
*λ* (male)	0.055	0.0092	0.16	0.21	0.10	0.37
*λ* (fem and fawn)	0.61	0.44	0.79	0.54	0.39	0.73
*D* (male km^−2^)	2.4	0.28	7.4	4.5	2.3	7.0
*D* (female km^−2^)	1.8	0.9	3.0	5.2	3.2	7.1
*D* (fawn km^−2^)	2.8	1.5	4.3	4.9	3.0	7.0
*D* (total km^−2^)	7.3	4.0	12.5	14.7	10.1	18.9
Adult sex ratio (*m*/*m + f*)	0.57	0.24	0.85	0.47	0.30	0.67
Fecundity	1.55	0.62	2.96	0.96	0.48	1.59

Note

The mean of the prior Gaussian distribution for *σ* for 2016 and 2017 was 305 m and 217 m, respectively, with a prior variance of 500 and 400, respectively. Encounter rates were assumed identical for females and fawns, but different for adult males. Adult sex ratio and fecundity are derived parameters calculated as the ratio of the number of adult males to adult females (i.e., 1 is equal sex ratio), and the number of fawns to adult females in the state space, respectively. Summary of 4,500 and 3,000 posteriors for 2016 and 2017, respectively.

**TABLE 4:** Parameter estimates of the spatial capture analysis where informative priors for the space use (*σ*) for females and fawns versus adult males were based on estimates published (Honzová, 2013) in the literature (SC‐lit)

	2016			2017		
	Estimate	Lower	Upper	Estimate	Lower	Upper
*σ* (male)	607	466	743	593	459	734
*σ* (fem/fawn)	154	114	219	170	146	196
*λ* (male)	0.046	0.0031	0.27	0.13	0.019	0.35
*λ* (fem and fawn)	0.59	0.42	0.78	0.53	0.38	0.72
*D* (male km^−2^)	0.84	0.093	4.2	0.60	0.063	2.1
*D* (female km^−2^)	5.1	2.5	7.9	5.2	2.9	7.7
*D* (fawn km^−2^)	7.1	3.9	8.4	4.9	2.8	7.6
*D* (total km^−2^)	13.4	7.0	18.0	11.0	6.9	15.1
Adult sex ratio (*m*/*m + f*)	0.15	0.01	0.48	0.10	0.01	0.31
Fecundity	1.3	0.74	2.3	0.97	0.44	1.60

Note

The prior mean of *σ* was 624 m for males and 287 m for females and fawns with variance of 5,000 and 1,000, respectively. Sex ratio and fecundity are derived parameters calculated as the ratio of the number of adult males to adult females (i.e., 1 is equal sex ratio), and the number of fawns to adult females in the state space, respectively. Summary of 4,500 and 3,000 posteriors for 2016 and 2017, respectively.

**TABLE 5:** Parameter estimates of the spatial capture–recapture analysis of antlered individually identified males combined with spatial capture of females and fawns (SC‐SCR)

	2016			2017		
	Estimate	Lower	Upper	Estimate	Lower	Upper
*p*_ID_	0.60	0.46	0.75	0.71	0.62	0.79
*σ* (male)	547	387	748	989	643	1521
*σ* (fem/fawn)	237	201	268	195	179	213
*λ* (male)	0.11	0.04	0.24	0.021	0.012	0.030
*λ* (fem/fawn)	0.64	0.46	0.82	0.47	0.32	0.64
*D* (male km^−2^)	0.6	0.3	1.1	2.0	1.8	2.6
*D* (female km^−2^)	2.5	1.1	4.1	5.4	3.0	8.1
*D* (fawn km^−2^)	3.8	2.0	6.2	5.4	3.2	8.6
*D* (total km^−2^)	7.0	4.5	10.4	13.0	9.0	17.4
Adult sex ratio (*m*/*m + f*)	0.20	0.10	0.40	0.27	0.18	0.40
Fecundity	1.57	0.64	2.97	1.02	0.49	1.67

Note

For females and fawns, the mean of the prior Gaussian distribution for *σ* for 2016 and 2017 was 305 m and 217 m, respectively, with a prior variance of 500 and 400, respectively. The parameter *p*_ID_ infers the probability a picture taken of an adult male could be individually identified. Space use and encounter rates were assumed identical for adult females and fawns. An informative prior for *σ* for adult females and fawns was used where the point value was set at the year‐specific sigma inferred by fDNA‐SCR (Table 1). Sex ratio and fecundity are derived parameters calculated as the ratio of the number of adult males to adult females (i.e., 1 is equal sex ratio), and the number of fawns to adult females in the state space, respectively. Summary of 3,000 posteriors for both years.

**FIGURE 4:**
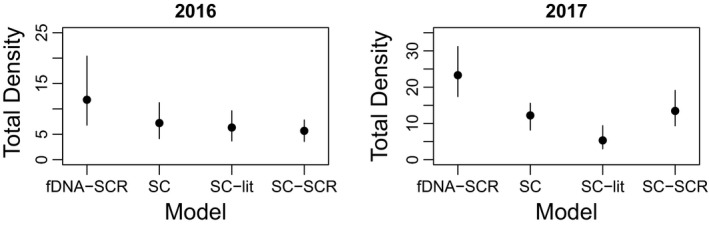
Estimates of total density (individuals/km^2^) in the study area plotted for both study years separately. The SCR analysis of fecal DNA (fDNA‐SCR) is reported in Table 1, SC analysis of wildlife camera data (SC) in Table 3, SC analysis based on literature estimate of sigma for adult females and fawns versus adult males (SC‐lit) is reported in Table 4 and the SC analysis of wildlife camera data of adult females and fawns combined with SCR analysis of individually identified pictures of adult males (SC‐SCR) is reported in Table 5. Dots denote the point estimate and lines the 95% credible interval

**FIGURE 5:**
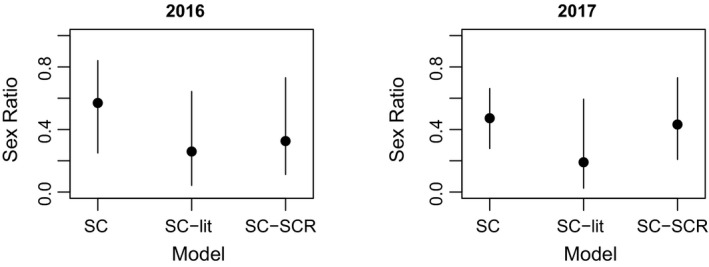
The derived estimates of sex ratio of adults expressed as density of adult males divided by the total density of both adult females and males in the study area for both study years separately. An equal adult sex ratio is hence 0.5 and values below 0.5 denote a female bias. The various SC models are reported in Tables 3, 4, 5. Note that the sex ratio inferred by the fDNA‐SCR (reported in Table 1) is based on both adults and fawns

The various SC approaches estimated, in general, a lower total density compared with fDNA‐SCR (Figure 4). In 2016, the credible intervals generally overlapped. In 2017, however, fecal DNA provided a higher estimate of density than the SC‐based estimates from wildlife camera pictures, except for the SC analysis that assumed male and female *σ* were identical to the *σ* estimated for fDNA (model “SC”) for which the credible intervals overlapped with the density inferred by fDNA‐SCR (Figure 4). Because the various SC approaches all assumed that *σ* for adult females and fawns were identical, the derived inference of fecundity was qualitatively the same across models (Tables 3, 4, 5). The adult sex ratio was equal in the SC model where the *σ* for adult males and females was assumed to be equal with the differences in the number of pictures taken of males and females attributed to a much lower encounter rate for adult males compared with adult females and fawns (Table 3; Figure 5). Strikingly, the SC approaches where adult male *σ* was allowed to differ from adult female *σ* (based either on literature values for *σ* or SCR analysis of individually identified male pictures) both inferred that the adult population was female‐biased as indicated by the 95% credible intervals CRI of adult sex ratio not including 0.5 (Tables 4 and 5; Figure 5).

## 4 DISCUSSION

In this study, we use an array of wildlife cameras taking pictures during a short time period (2–3 weeks) in late summer just prior to harvest. Our main finding is that wildlife cameras indeed collect a sufficient amount of information to allow calculating the population parameters of interest, but that they may provide different inferences than the fecal DNA‐based analyses. Firstly, based on fecal DNA the space use of males and females is not significantly different. This result is surprising as sex differences in movement of male and female adult white‐tailed deer are well established (Dechen Quinn et al., 2013; Honzová, 2013; Lesage et al., 2000). Because DNA cannot be used to distinguish age groups, sexes in analyses based on fecal DNA refer to both fawns and adults. The space use inferred here by SCR analysis of fecal DNA agrees well with that found in an earlier study conducted in a different area using fecal DNA in late summer (*σ* 190 m; Poutanen et al., 2019). The *σ* (for both sexes) inferred by using fecal DNA (305 m in 2016 and 217 m in 2017) agrees well with what is expected on the basis of published home‐range area of GPS collar located white‐tailed deer adult females in Finland (287 m; Honzova et al., 2013; see Material and Methods for calculation of *σ*). However, our SCR analysis of pictures of individually identified adult males concludes that adult males have a *σ* that is about threefold the *σ* inferred by SCR of fecal DNA, a finding much in line with literature values. However, we cannot infer *σ* of adult females using wildlife cameras as adult females cannot be individually identified. Taken together, nevertheless, it could be that fecal DNA‐based SCR does not capture the heterogeneity in *σ* between adult males and adult females in cases as ours where the preharvest population is studied and a substantial part of the population consists of fawns that have a *σ* more comparable to adult females than adult males.

Secondly, the total density inferred by fecal DNA is in general higher than the density inferred by SC analysis of wildlife camera data, and in one of the two study years (2017) significantly higher than two of the three SC models. While our a posteriori simulations show that the fDNA‐based estimate of density in 2016 risks being an overestimate, bias for 2017 is largely absent. Finding that SC analyses provide lower estimates of density than fDNA‐based SCR is surprising as simulations show that, all else being equal, SC analyses tend to overestimate density and underestimate space use (Royle, Richard, et al., 2013) and are generally imprecise (Augustine et al., 2019). At the same time, the SC model has clear potential for inferring density of wildlife species based on a variety of relatively simple methods including cost‐effective approaches for recording presence/absence (Ramsey et al., 2015). Importantly, density and space use are inversely related (Efford et al., 2016) and with lack of information on how individuals move, an SC model's conservative inference of space use typically leads to large density. We indeed find that our SC models typically infer *σ* to be below the informative prior for *σ*, and thus are unlikely to infer density conservatively. We find that assuming literature values of *σ*, which is typically the only option (Chandler & Royle, 2013), provides particularly low estimates for total density, and is hence from that perspective most conservative. However, as a corollary, literature‐based prior for *σ* also infer the most female‐biased adult sex ratios and are hence not conservative from that perspective. A further, partially related, issue is that our DNA sampling design may be suboptimal to detect individuals moving larger distances. Based on *σ* provided by fDNA‐SCR, which is same for adult males, adult females, and fawns (305 m in 2016 and 217 m in 2017), our fecal DNA clusters are at distances of what Sun et al. (2014) suggested for an optimal sampling design (intercluster spacing maximally two times *σ*). Nevertheless, the wildlife cameras were placed at the same distances as DNA clusters but they still allow SCR analysis of adult males (*σ* based on cameras 547 m in 2016 and 989 m in 2017). One explanation for higher densities based on fecal DNA is therefore that DNA sampling captures primarily shorter movement such as of adult females with fawns, and probably adult males when moving at shorter distances. DNA captures are based on feces left on the ground and cameras take pictures when they detect moving individuals. Somehow related to this aspect, these two approaches may detect different movement behaviors of adult males. Hence, an important improvement in future studies would be to have spacing of fDNA and camera traps both at short and longer distances in order to better capture the heterogeneity in *σ* across age/sex classes.

An exciting aspect of the theory underlying spatial capture–recapture models is that certain (derived) parameters are assumed to be identical across model formulations. In particular, the parameter *σ* describing space use as well as the number and location of the activity centers of individuals are assumed to be the same (Kéry & Royle, 2020; Royle, Richard, et al., 2013). There are, however, not many studies directly comparing inferences obtained using different SCR methodologies (but see, e.g., Burgar et al., 2018). One advantage of having SCR parameters in common is that data of different sources (e.g., fecal DNA and wildlife cameras, or fecal DNA and radio‐tracking data) can be combined (e.g., Gopalaswamy et al., 2012), although nonindependence of detections across methods needs to be carefully considered (Clare et al., 2017). Combination of spatial capture–recapture data from DNA and from cameras typically provide more precise estimates (Burgar et al., 2018; Sollmann, Gardner, et al., 2013; Sollmann, Tôrres, et al., 2013). Other studies have focused on developing “hybrid” approaches with models explicitly combining count and SCR data, because count data are easier and cheaper to obtain as it does not require individual identification. For example, DNA‐based spatial capture–recapture has been combined with N‐binomial mixture modeling of data from single cameras for estimation of ungulate density over landscape scale combining information from several sites (Furnas et al., 2018). Chandler and Clark (2014) showed that SC data collected in some years combined with SCR data collected in other years are a cost‐effective approach to improve temporal monitoring. Jiménez et al. (2019) developed an approach to integrate information on identified individuals with information on nonidentified ones and showed this integration to be particularly important in low‐density populations. A challenge in combining the fDNA and wildlife camera data we collected in this paper is that these two methods do not identify the same age classes. Future work, for example, using partial identity models (Augustine et al., 2018), could lead to potential fruitful types of hybrid SCR models for this kind of data, leading to improved inference of density, sex ratio, and fecundity.

### 4.1 Population parameters inferred by wildlife cameras

The regional density of the preharvest population of white‐tailed deer in 2016 and 2017 was 3.9 and 4.2 white‐tailed deer per km^2^, respectively (Riistaweb, 2021). In comparison, the density estimates we obtain here are larger. Apart from very different methodologies underlying these density estimates, it is likely that local white‐tailed density in our study area is particularly high compared with the regional average. The current challenge for wildlife managers is to keep the white‐tailed deer population, which has dramatically increased in the last decade, at a sustainable level. To this end, improving the quality and the spatial resolution of estimates of white‐tailed deer density likely is key. Our findings demonstrate that wildlife cameras allow inferences of density, but also that the uncertainty in these density estimates is substantial. Therefore, a possible use of wildlife camera data for wildlife management is to provide additional and independent information on local population density, fecundity, and sex ratio to be integrated with larger‐scale population dynamical modeling based on, for example, hunting statistics.

Wildlife camera data suggest that adult sex ratios in the white‐tailed deer population may be female‐biased; a female bias is evident in both study years in the SC models that allow space use to differ between adult males and adult females. It is clear already from the raw data that more adult white‐tailed deer females and fawns are counted compared with adult males, and this sex bias persists despite the model accommodating that adult white‐tailed deer males have a much lower encounter rate than females and fawns. In fact, also the fDNA data show evidence of a female‐biased sex ratio in 2017, which reflects adult sex ratio as fawns are produced at equal sex ratio. One explanation is that our study area happens to contain more adult females than adult males, while the white‐tailed deer population at a larger geographic scale has an equal sex ratio. Our study area could, for example, be particularly attractive to females and their offspring during early autumn when we conducted this study. This could be due to some resources the area provide, for example, cover (Kie & Bowyer, 1999). On the other hand, a female‐biased adult sex ratio in the overall population can be partly due to the fact that hunting regulations (at the time of our study) specify that an adult female with offspring cannot be harvested, whereas males lack such a “life insurance” possibly leading to a higher risk for an adult male of being harvested compared with an adult female. For example, Kekkonen et al. (2016) showed that harvested adult females are older than adult white‐tailed deer males, which is consistent with a higher mortality rate for white‐tailed deer males in Finland. Further research into the adult sex ratio of white‐tailed deer in Finland is needed to improve our understanding of this important demographic parameter in this heavily harvested population.

A wildlife camera study prior to harvest in late summer allows inference of fecundity as adult females and fawns are readily distinguished. It is, at least in the middle of summer, possible to use individual‐specific pattern of the spotted pelage of fawns to construct individual‐based SCR models (Chandler et al., 2018). Our analysis of camera data using the SC approach is limited by the assumption that detection and space use of adult females and fawns are identical. This assumption is necessary as we have no information to differentiate between these age classes. Hence, the inferred fecundity is simply the ratio of fawn pictures over adult female pictures (where the SC approach of course provides a measure of uncertainty). At the same time, the model assumes that activity centers of fawns are independent of their mothers and of each other. While fawns in late summer are presumably semi‐independent of their mother, it is likely that the activity centers of a mother and her fawns are close. This kind of nonindependence likely causes no bias for inferring density (Bischof et al., 2020; Russell et al., 2012), but a generally applicable way to accommodate dependencies of home ranges of parents and their offspring has not been developed (Bischof et al., 2020). White‐tailed deer commonly have two fawns, and also triplets are observed. Although fecundity of one‐year‐old white‐tailed deer females is low (Ryman et al., 1981), females aged 2–6 produce 1.3–1.5 offspring per female. After these ages, a decline in reproductive output commences. Fecundity values of 1.3–1.7 (point estimates for 2016) are from that perspective on the high side, whereas a fecundity of around 1 (point estimates for 2017) appears more reasonable. On the other hand, the harvesting rate of white‐tailed deer is substantial, and it is likely that a large fraction of the population consists of young females (cf Kekkonen et al., 2016) with high reproductive potential.

## 5 CONCLUSION

The number of wildlife cameras in use rapidly increases, doubling in number approximately every 3 years (Burton et al., 2015). This development creates a powerful incentive to wildlife managers and researchers to—through citizen science type of effort—obtain potentially valuable information on population parameters of wildlife. However, to convert pictures into population biological information, a number of analysis steps are required, both in terms of interpreting pictures and in terms of analyzing these interpretations. We here focused on spatial capture (SC) analysis using a single (relatively large) array of wildlife cameras deployed during a short period of time (2–3 weeks) in late summer. SC requires minimal picture interpretation (count per picture), and we show it can infer density, adult sex ratio, and fecundity in the preharvest population of the white‐tailed deer. Although SC in general is considered to risk overestimation of density, we find that this approach provides an estimate of total density that is conservative when compared to density estimated using fecal DNA in a spatial capture–recapture context. The main disadvantage of the SC approach is that it often requires prior or additional information on at least one SCR parameters. We here show that a literature‐based informative prior of space use (*σ*) provides comparable although potentially most conservative (in terms of total density) estimates in relation to year‐specific *σ* inferred from fDNA and/or SCR of wildlife camera data (for adult males). A spatial capture scheme of wildlife camera data therefore has potential to be a source of population biological information of relevance to wildlife management.

## CONFLICT OF INTEREST

The authors declare they have no conflicting interest.

## AUTHOR CONTRIBUTIONS

**Jon E. Brommer:** Conceptualization (equal); Formal analysis (lead); Funding acquisition (equal); Methodology (lead); Project administration (lead); Writing‐original draft (lead); Writing‐review & editing (equal). **Jenni Poutanen:** Conceptualization (equal); Formal analysis (equal); Investigation (equal); Methodology (equal); Writing‐review & editing (equal). **Jyrki Pusenius:** Funding acquisition (equal); Investigation (equal); Project administration (equal). **Mikael Wikström:** Funding acquisition (equal); Project administration (equal); Writing‐review & editing (equal).

## Data Availability

The fDNA data for spatial capture–recapture as well as the wildlife camera data for the various spatial capture models and the script for these spatial capture models is available from the repository Dryad https://doi.org/10.5061/dryad.dz08kprz8.
